# Supplementary figures and images for: The decrotonylase FoSir5 facilitates mitochondrial metabolic state switching in conidial germination of Fusarium oxysporum
Source: eLife. 2021 Dec 20;10:e75583. doi: 10.7554/eLife.75583 (PMC8730727; doi:10.7554/eLife.75583)

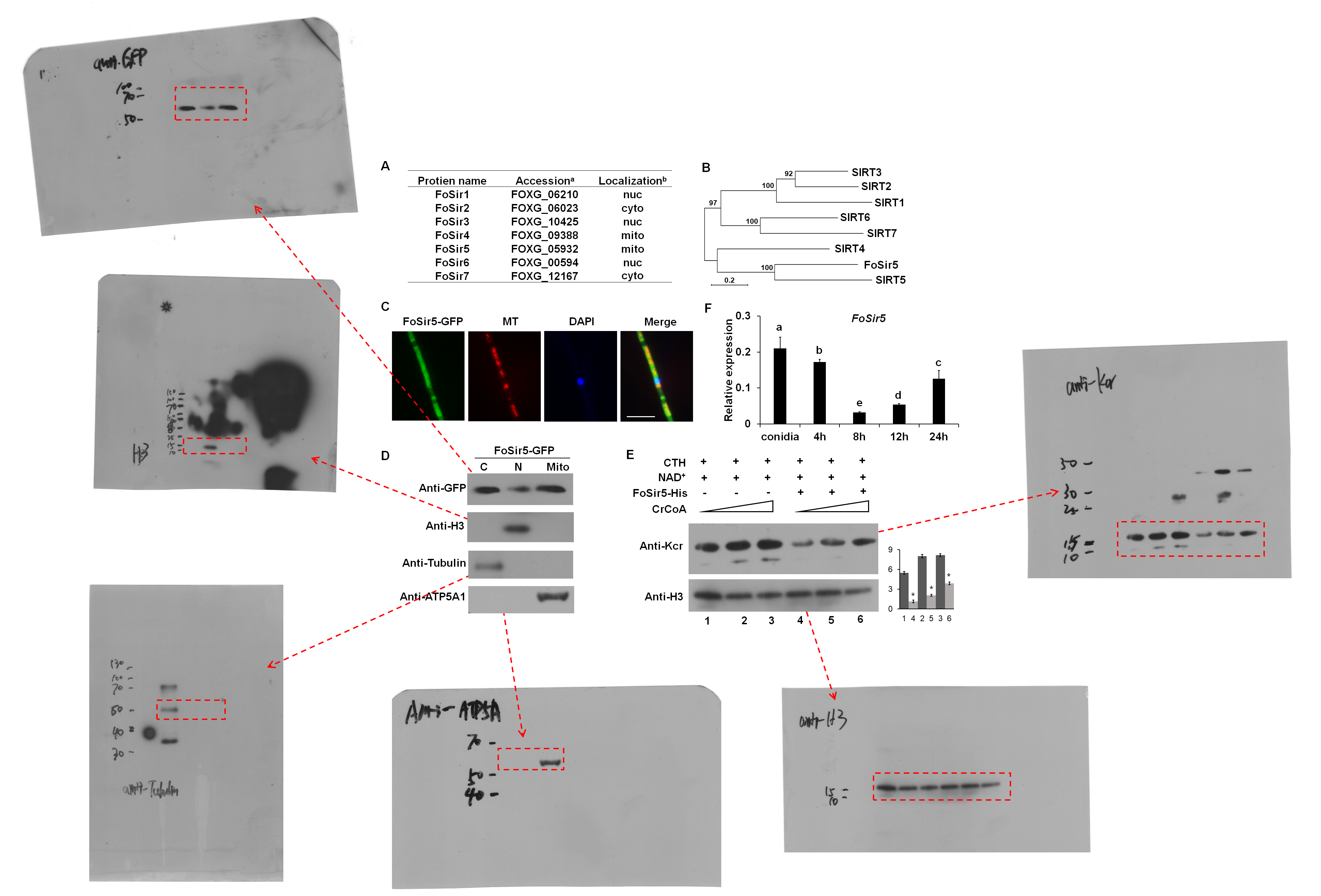

Supplement: Figure 1—source data 1. — (A) Sirtuin proteins in F. oxysporum with predicted subcellular localizations. aAccession number of the full-length protein sequence available at Ensembl. bLocalization of the F. oxysporum Sir2 protein determined by WoLF PSORT. (B) Phylogenetic tree relating FoSir5 to the orthologous human Sirtuin isoforms SIRT1 (NP_036370), SIRT2 (NP_085096), SIRT3 (NP_001357239), SIRT4 (NP_036372), SIRT5 (NP_001363737), SIRT6 (NP_057623), and SIRT7 (NP_057622). The tree is based on neighbor-joining analysis using MEGA-X. (C) Fluorescence microscopy analysis of FoSir5-GFP localization with MitoTracker Red (MT) and DAPI. Scale bars = 10 µm. (D) Subcellular fractionation of FoSir5-GFP transformants in F. oxysporum. Nuclear, cytoplasmic, and mitochondrial proteins were separately extracted and FoSir5-GFP were detected with anti-GFP antibody (Materials and methods). The fractionation controls were ATP5A1 (mitochondria), tubulin (cytosol), and histone H3 (nucleus). C, cytosol; N, nucleus; Mito, mitochondria. (E) In vitro Kcr assays with 50 µg of native calf thymus histone (CTH), 5 mM NAD+, and 0.5 µg of FoSir5-His in the presence of 50, 100, or 200 µM crotonyl-CoA. Reaction materials were analyzed by Western blotting with anti-Kcr or anti-H3 antibody. Each scale bar represents the mean ± SD for triplicate experiments. * indicates a significant difference between different pairs of samples (p < 0.05). (F) Expression profile of FoSir5 in conidia, mycelium, and during the germination process. The expression levels were normalized to that of the F. oxysporum elongation factor one alpha (EF-1α) gene. The presence of different letters above the mean values of three replicates indicates a significant difference between different samples (p < 0.05, ANOVA). (The red arrow indicates the original SDS–PAGE gels that were cropped for this panel.) [file elife-75583-fig1-data1.zip › Figure 1-source data.tif]

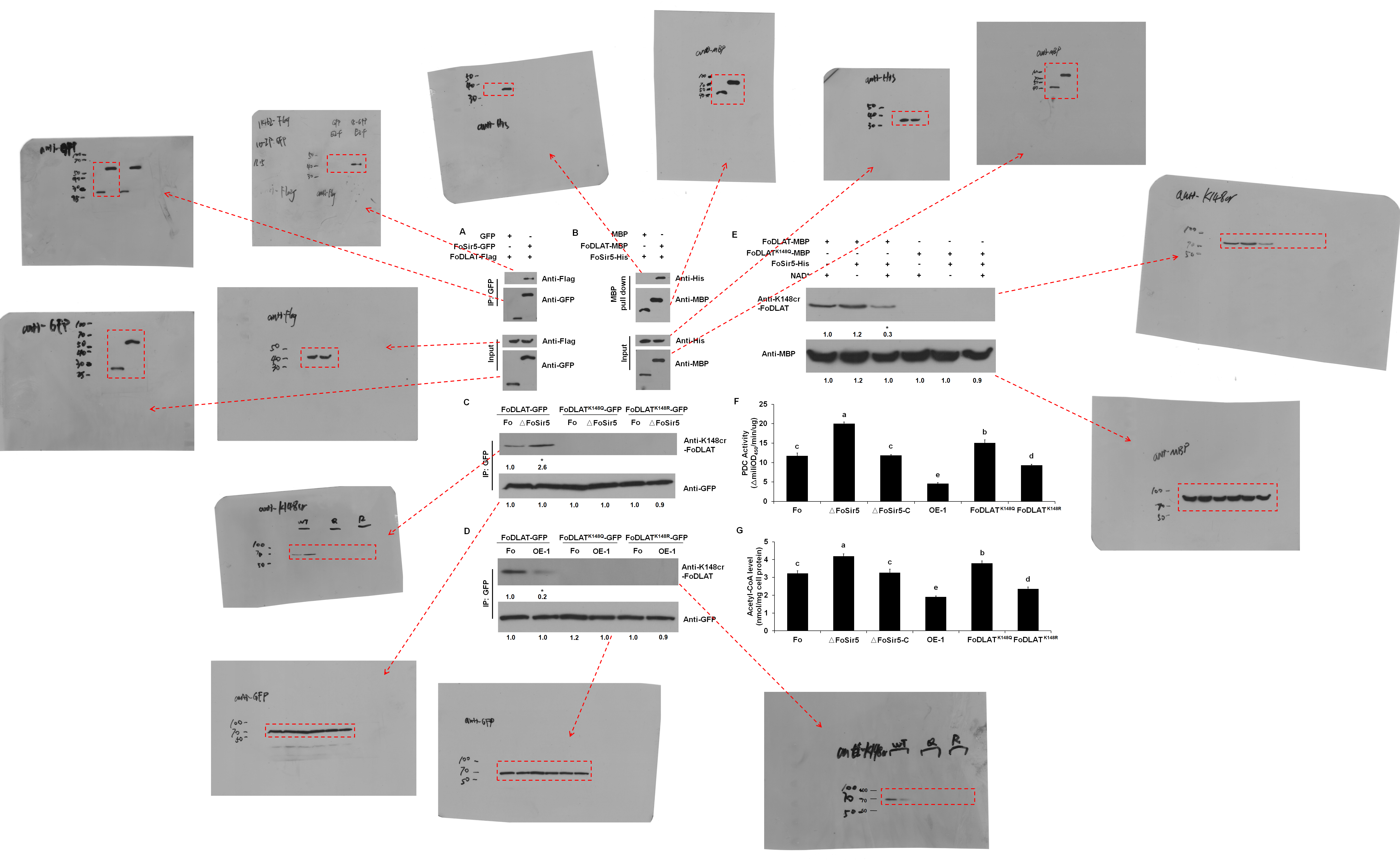

Supplement: Figure 2—source data 1. — (A) Co-IP assays reveal physical interaction of FoSir5-GFP and FoDLAT-Flag. Western blot analysis of cell extracts from transformants co-expressing FoDLAT-Flag with GFP or FoSir5-GFP and elution from anti-GFP agarose. The fusion proteins were detected with anti-Flag or anti-GFP antibody. (B) In vitro pull-down assays to detect FoSir5-His with MBP or the FoDLAT-MBP fusion protein. FoDLAT-MBP was used as bait to pull down the FoSir5-His protein from the induced cell extracts. The MBP protein was assayed as a negative control. Input and bound forms of the pull-down fractions were detected with anti-His or anti-MBP antibody. (C–D) The K148 crotonylation (anti-K148cr-FoDLAT, top panel) and amount (anti-GFP, bottom panel) of FoDLAT-GFP and its mutant isoforms in the ΔFoSir5 (C) and OE-1 strain (D). Proteins were immunoprecipitated with anti-GFP antibody agarose beads and analyzed by anti-K148cr-FoDLAT or anti-GFP antibody. Representative gels are shown from experiments carried out at least twice. Numbers below the blots represent the relative abundance of K148-crotonylated FoDLAT. Anti-GFP immunoblotting was used to show equal loading. (E) FoSir5 directly decrotonylates FoDLAT in vitro. Purified FoDLAT protein or its K148Q isoform (50 ng) were incubated with or without 50 ng of purified FoSir5 in the absence or presence of 5 mM NAD+ and then analyzed by immunoblotting using anti-K148cr-FoDLAT or anti-His antibody. Each gel shown is representative of two experiments. Numbers below the blots represent the relative abundance of K148-crotonylated FoDLAT. Anti-MBP immunoblotting was used to show equal loading. (F–G) FoSir5 and K148 mutant FoDLAT affected pyruvate dehydrogenase complex (PDC) activity (F) and acetyl-CoA production (G) in F. oxysporum. PDC activity and acetyl-CoA production were determined in germinating conidia at 8 hr. The presence of different letters above the mean values of three replicates indicates a significant difference between different strains (Pp< 0. [file elife-75583-fig2-data1.zip › Figure 2-source data.tif]

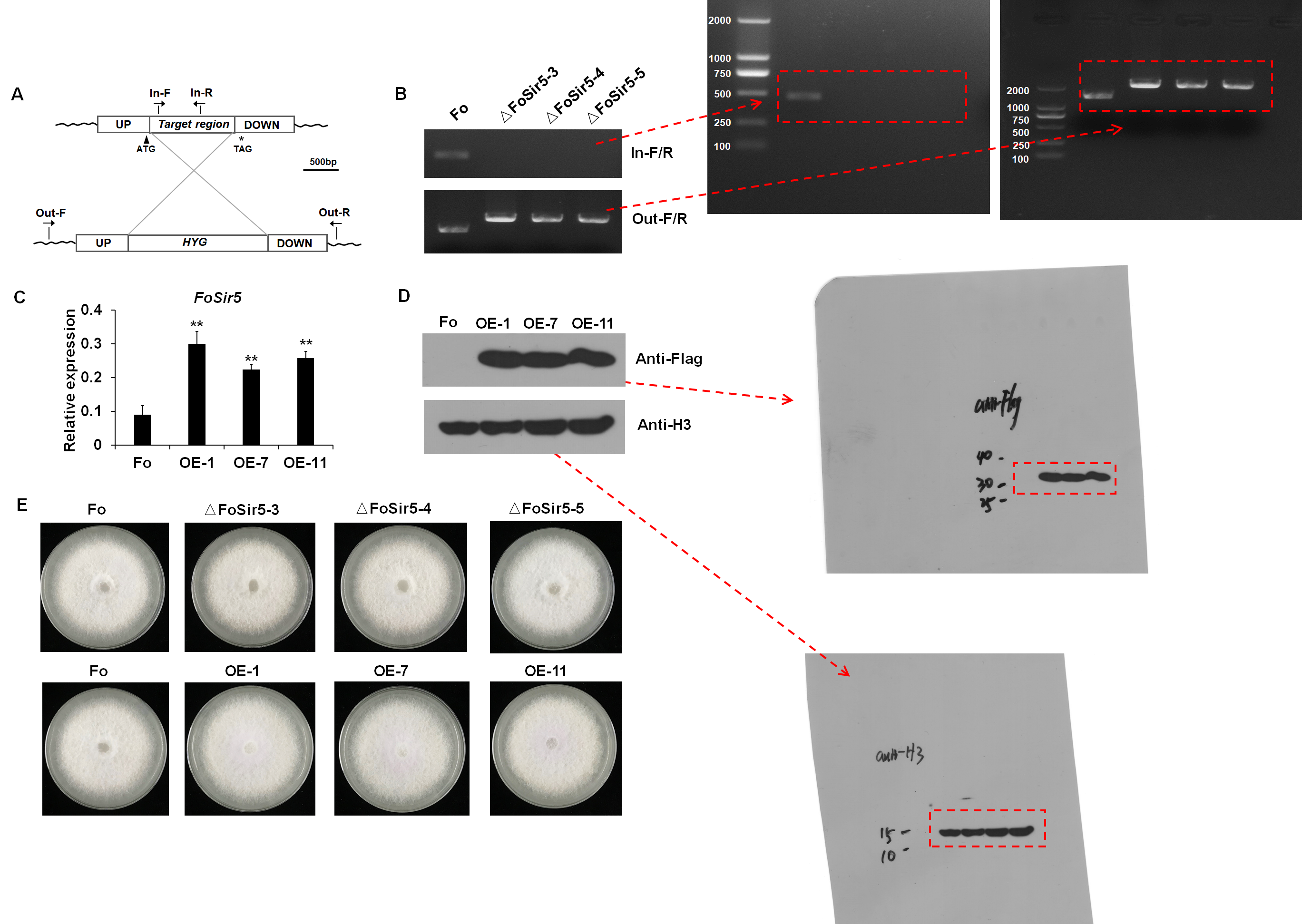

Supplement: Figure 2—figure supplement 2—source data 1. — (A) Schematic representation of the targeted deletion of FoSir5. (B) PCR analysis of targeted deletion in the ΔFoSir5 strains. Genomic DNA was analyzed by PCR with the primer pairs indicated in panel (A). (C–D) Real-time (RT)-PCR (C) and Western blotting (WB) (D) analysis of the FoSir5-Flag-overexpression transformants. Data of RT-PCR are the means ± SDs (n = 3); **p < 0.05 by unpaired two-tailed t-test. (E) Mycelial growth of the indicated strains on potato dextrose agar (PDA) plates after 3 days of cultivation. (The red arrow indicates the original AGE or SDS–PAGE gels that were cropped for this panel.) [file elife-75583-fig2-figsupp2-data1.zip › Figure 2-figure supplement 2-source data.tif]

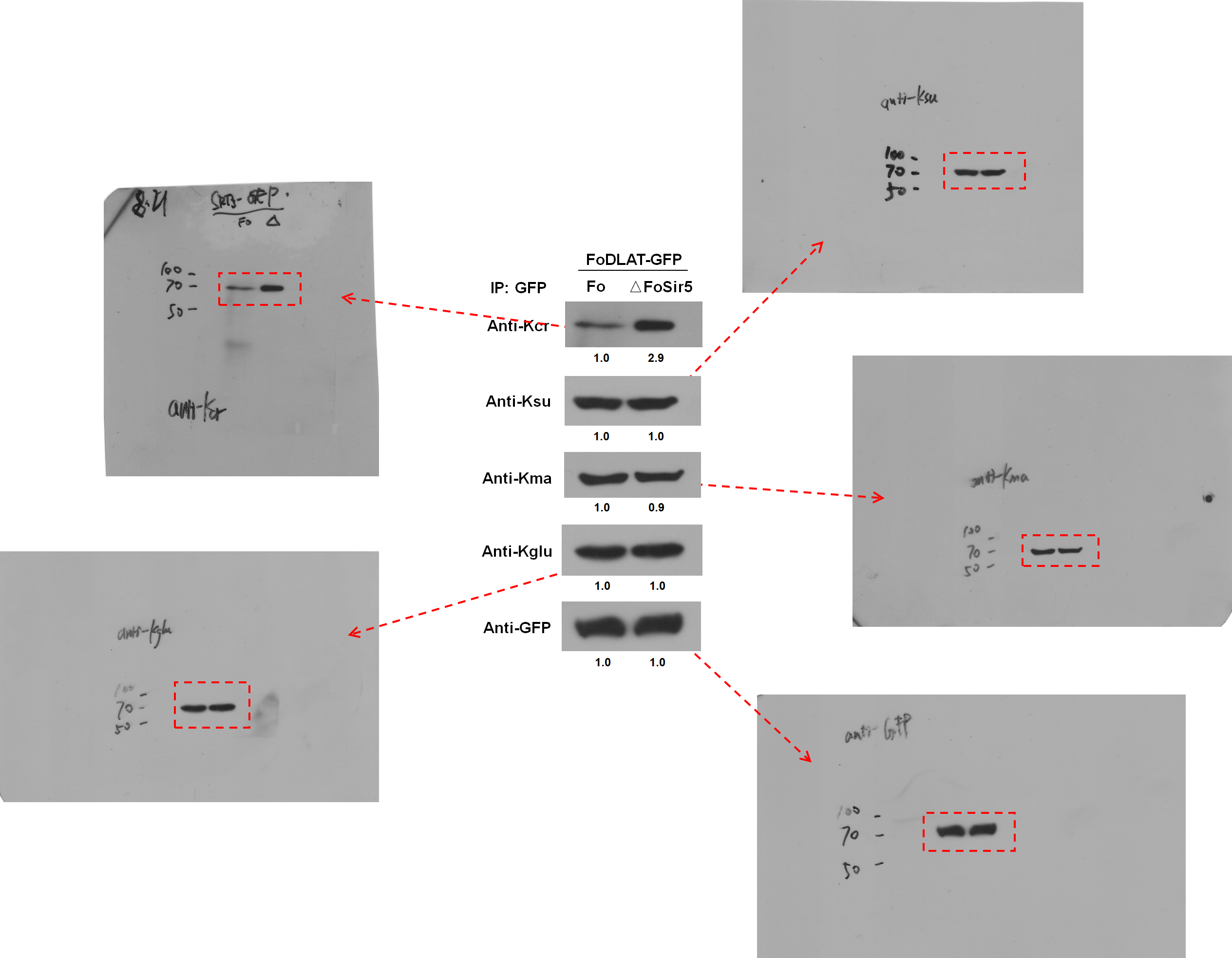

Supplement: Figure 2—figure supplement 3—source data 1. — Proteins were immunoprecipitated with anti-GFP antibody agarose beads and analyzed by Western blot using the indicated antibodies. Representative gels are shown from experiments carried out at least twice. Anti-GFP immunoblotting was used to show equal loading. (The red arrow indicates the original SDS–PAGE gels that were cropped for this panel.) [file elife-75583-fig2-figsupp3-data1.zip › Figure 2-figure supplement 3-source data.tif]

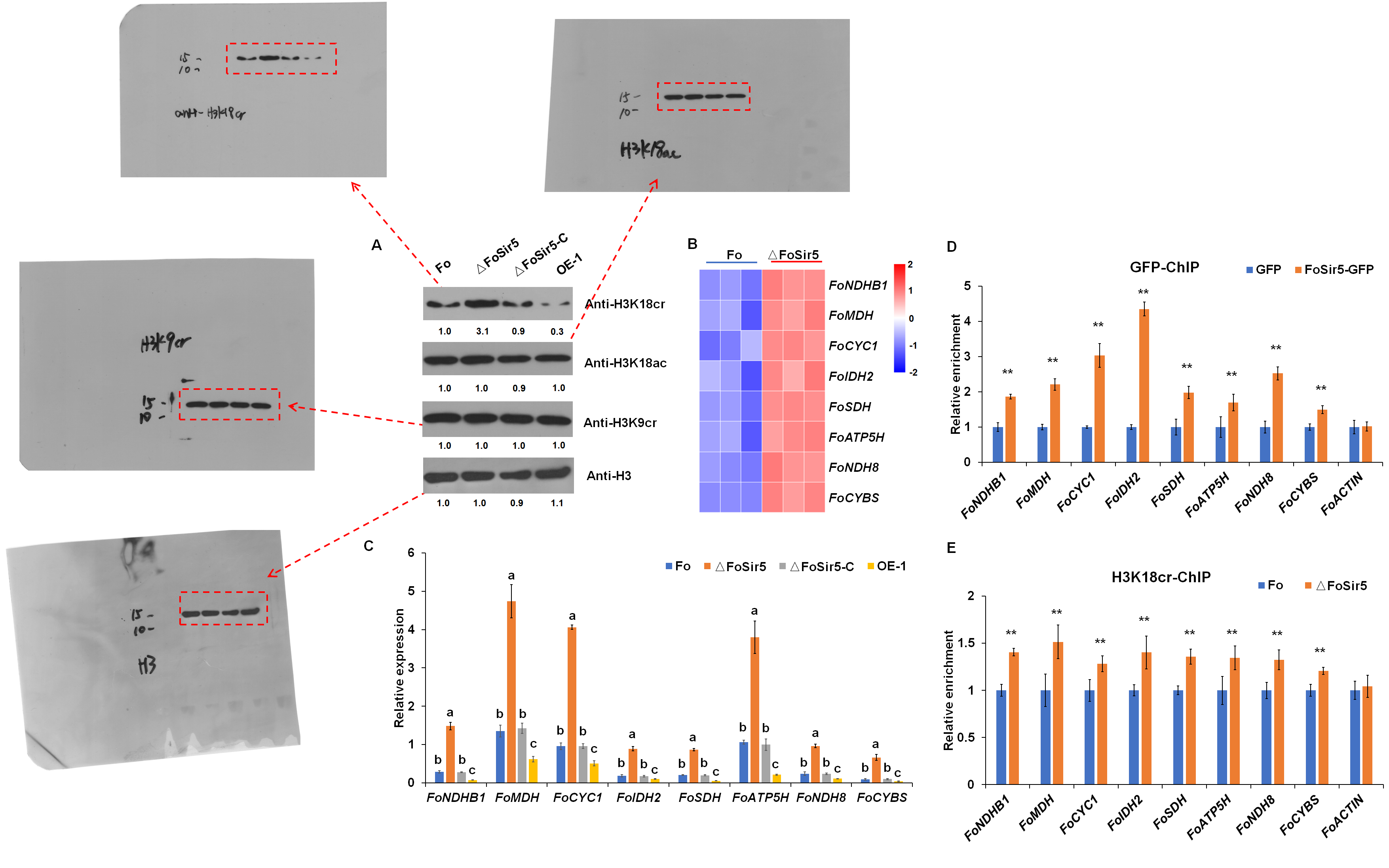

Supplement: Figure 3—source data 1. — (A) Western blot analysis showed the effect of FoSir5 on histone H3K18 crotonylation and acetylation, and histone H3K9 crotonylation using the indicated antibodies. Numbers below the blots represent the relative abundance of different modifications. Anti-H3 immunoblotting was used to show equal loading. (B) RNA-seq analysis of eight upregulated genes involved in aerobic respiration including NDHB1 (NADH-quinone oxidoreductase chain B 1), MDH (malate dehydrogenase), CYC1 (cytochrome C1), IDH2 (isocitrate dehydrogenase subunit 2), SDH (succinate dehydrogenase), ATP5H (ATP synthase D chain), NDH8 (NADH dehydrogenase iron-sulfur protein 8), and CYBS (succinate dehydrogenase cytochrome b small subunit). Differential expression in three biological replicates illustrated using a heat map with colored squares indicating the range of expression referred to as the FPKM value. (C) qRT-PCR validation of aerobic respiration-related genes in the indicated strains. The letters above the mean values of three replicates indicate significant differences between different strains (p < 0.05, ANOVA). (D–E) Relative enrichment of the immunoprecipitated promoter regions in aerobic respiration-related genes determined using anti-GFP antibody in the FoSir5-GFP strain and Fo strain containing GFP alone (D) or using anti-H3K18cr antibody in the Fo and ΔFoSir5 mutant strains (E). The fold enrichment was normalized to the input and internal control gene (β-tubulin). Data are the means ± SDs (n = 3); **p < 0.05 by unpaired two-tailed t-test. (The red arrow indicates the original SDS–PAGE gels that were cropped for this panel.) [file elife-75583-fig3-data1.zip › Figure 3-source data.tif]

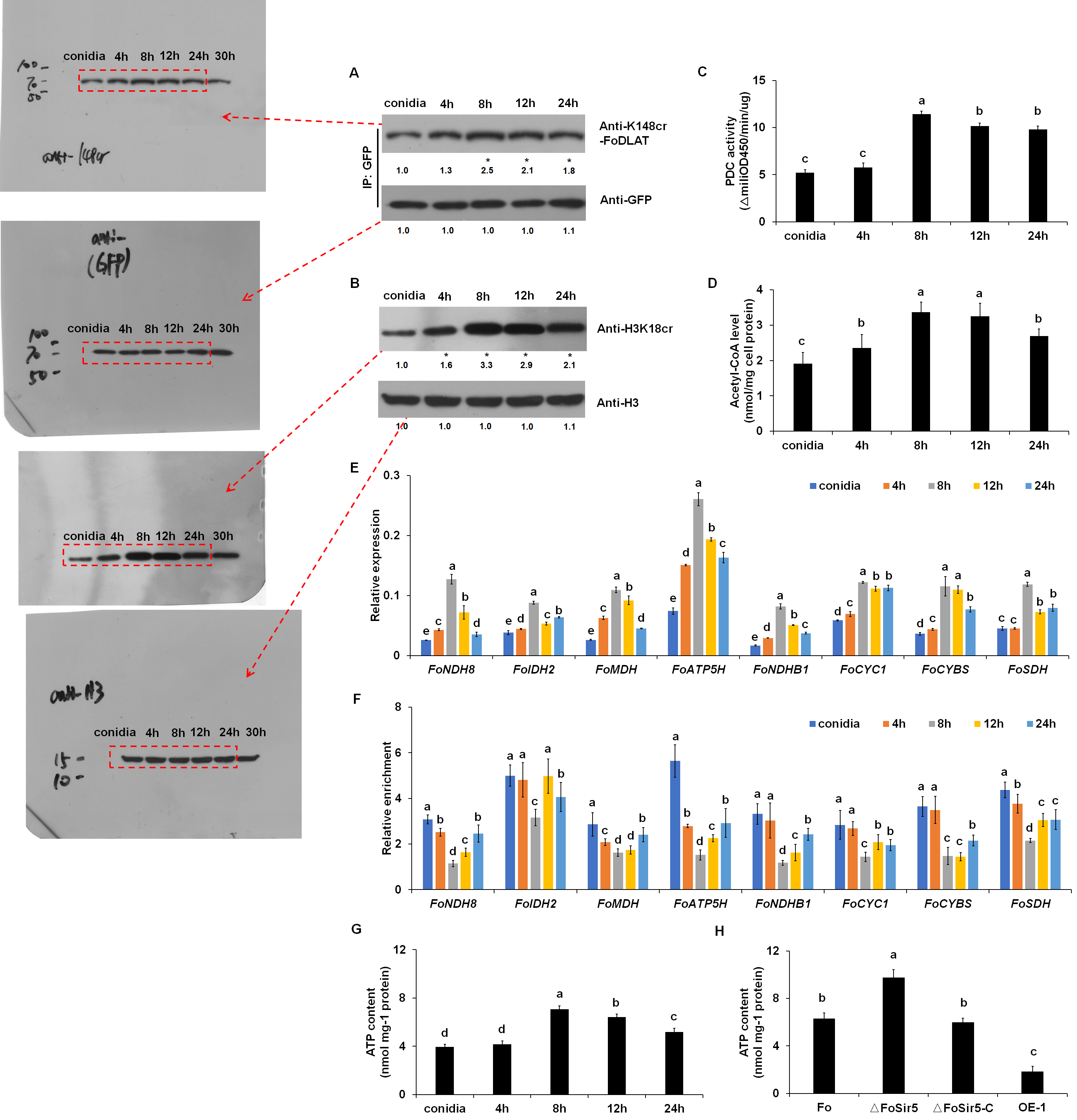

Supplement: Figure 4—source data 1. — (A–B) Western blot analysis showed the dynamic changes of FoDLAT K148 (A) and histone H3K18 (B) crotonylation during germination using the indicated antibodies. Numbers below the blots represent the relative abundance of FoDLAT-K148cr or H3K18cr. Anti-GFP or anti-H3 immunoblotting was used to show equal loading, respectively. (C–D) Pyruvate dehydrogenase complex (PDC) activity (C) and acetyl-CoA production (D) in F. oxysporum during germination were determined. (E) Expression profile of the aerobic respiration-related genes during the germination process. (F) Relative enrichment of the immunoprecipitated promoter regions in aerobic respiration-related genes during germination determined using anti-GFP antibody in the FoSir5-GFP strain driven by the native promoter. The fold enrichment was normalized to the input and internal control gene (β-tubulin). (G) ATP content of F. oxysporum during germination. (H) Effect of FoSir5 on the ATP content of the indicated strains, as determined in germinating conidia at 8 hr post incubation (h.p.r.). The presence of different letters (A–H) above the mean values of three replicates indicates a significant difference between different samples (p < 0.05, ANOVA). (The red arrow indicates the original SDS–PAGE gels that were cropped for this panel.) [file elife-75583-fig4-data1.zip › Figure 4-source data.tif]
